# Evaluation of the Role of Intravenous Lidocaine Infusion in the Management of Chronic Pain: A Retrospective Study

**DOI:** 10.1155/prm/1671698

**Published:** 2026-06-27

**Authors:** Abdelhady S. M. Ali, Thanthullu Vasu, Pradeep Ingle

**Affiliations:** ^1^ Anaesthesia and Chronic Pain Department, University Hospitals of Leicester NHS Trust, Leicester, UK, nhs.uk; ^2^ Anaesthetic and Chronic Pain Department, Assiut University Hospitals, Asyut, Egypt, aun.edu.eg

**Keywords:** chronic myofascial pain, fibromyalgia, lidocaine infusion, neuropathic chronic pain

## Abstract

**Background:**

Intravenous lidocaine infusion is increasingly used in chronic pain services; however, real‐world data regarding durability of analgesic benefit and safety in routine practice remain limited.

**Objective:**

To evaluate patient‐reported analgesic outcomes, duration of benefit and safety profile of intravenous lidocaine infusion in a tertiary chronic pain service.

**Methods:**

A retrospective service evaluation was conducted at Leicester General Hospital, pain management department, including all adult patients who received intravenous lidocaine infusion between October 2024 and March 2025. Lidocaine was administered at 3 mg/kg over 1 h under monitored day‐case conditions. Patients were contacted by structured telephone follow‐up to assess Numerical Rating Scale (NRS) pain scores, duration of benefit, adverse effects and satisfaction. Descriptive statistics were used to summarise outcomes.

**Results:**

Of 136 treated patients, 112 completed follow‐up (82.4%). Mean baseline NRS decreased from 8.0 to 4.5 during the early posttreatment period, representing an absolute reduction of 3.5 points. More than 60% of patients reported clinically meaningful improvement, while sustained pain relief beyond 3 months was reported by 29% of patients. Adverse effects occurred in 17% of cases and were mild and self‐limiting, with no serious adverse events observed. Overall satisfaction with treatment was high, with 85% of patients indicating that they would recommend intravenous lidocaine infusion for chronic pain management. Among these, 47% were receiving the infusion for the first time.

**Conclusion:**

In this real‐world cohort, intravenous lidocaine infusion was associated with short‐ to medium‐term patient‐reported pain improvement and a favourable safety profile. While a subset experienced sustained benefit beyond 3 months, most patients reported shorter duration relief. Prospective controlled studies using validated outcome measures are required to define long‐term efficacy and optimal patient selection.

## 1. Introduction

Lidocaine, an amide type local anaesthetic, has traditionally been used for regional anaesthesia and arrhythmia management. Beyond its local anaesthetic properties, lidocaine exhibits systemic analgesic and anti‐inflammatory effects. Intravenous lidocaine (IVL) infusion has increasingly been applied in day‐case pain settings for conditions including fibromyalgia, neuropathic pain, trigeminal neuralgia, chronic radicular pain and chronic myofascial pain. Its proposed mechanisms include inhibition of voltage‐gated sodium channels and NMDA receptors, modulation of G protein coupled receptor pathways and suppression of spontaneous ectopic discharges arising from injured peripheral nerves and dorsal root ganglion neurons. These effects may attenuate central sensitisation and abnormal neuronal excitability, which are key contributors to neuropathic and deafferentation‐related pain states [1–6].

Clinical evidence supporting IVL in chronic pain is heterogeneous and condition specific. Randomised controlled trials and systematic reviews have demonstrated short‐term analgesic benefit compared with placebo in several neuropathic pain conditions, including peripheral nerve injury, diabetic neuropathy, postherpetic neuralgia, trigeminal neuralgia and synesthetic pain following spinal cord injury [4, 5, 7–9]. Response rates appear highest in radicular and peripheral neuropathic pain syndromes. In contrast, outcomes have been inconsistent in sympathetically mediated pain and cancer‐related pain syndromes. Studies in complex regional pain syndrome suggest that multiday escalating infusions targeting therapeutic plasma concentrations may provide analgesia lasting several months, particularly for mechanical and thermal allodynia. However, across most studies, analgesic benefit is often transient, with limited data regarding sustained long‐term outcomes [6, 10–12].

Safety data from controlled settings suggest that IVL is generally well tolerated when administered with appropriate monitoring. Reported adverse effects include light‐headedness, nausea, perioral numbness, sedation and dizziness, with serious complications such as seizures or arrhythmias being rare under monitored conditions. Nevertheless, dose‐related toxicity remains a concern, and there are limited real‐world audit data describing complication rates and long‐term follow‐up in routine clinical practice [9, 13–15].

Despite increasing use of IVL infusions in chronic pain services, published audit data remain sparse, particularly regarding durability of analgesic response and safety in standard outpatient protocols. Furthermore, the mixed and condition‐specific efficacy observed in existing trials highlights the need to better characterise patient selection, duration of benefit and adverse event profiles in real‐world cohorts. Long‐term follow‐up data may represent an important strength in understanding the clinical utility of this intervention beyond short‐term experimental settings.

The primary aim of this study was to evaluate the efficacy of IVL infusion in the management of chronic pain in a routine clinical setting. Secondary aims were to assess the frequency and nature of adverse effects associated with treatment and to evaluate the duration of analgesic benefit following infusion.

## 2. Methods

### 2.1. Study Design

This was a retrospective study registered with the University Hospitals of Leicester Trust Clinical Governance Department (under No. 14285).

### 2.2. Setting and Participants

All consecutive adult patients who received IVL infusion at the Day Theatre, Leicester General Hospital, between October 2024 and March 2025 were included. The IVL service operates within a consultant‐led tertiary chronic pain service at University Hospitals of Leicester NHS Trust and is delivered as a monitored day‐case intervention.

### 2.3. Clinical Protocol for IVL Administration

Patients were initially assessed in the chronic pain clinic by a consultant in pain medicine. A comprehensive clinical evaluation was undertaken, including pain history, examination, prior treatment response, medication review and multidisciplinary input where appropriate. IVL was offered to selected patients with refractory chronic pain after failure of conventional pharmacological and nonpharmacological therapies.

### 2.4. Indications

IVL was considered in adult patients aged 18 years or older with chronic pain of more than 6‐month duration, particularly in the context of

• Widespread chronic pain, including fibromyalgia
• Chronic myofascial pain
• Chronic neuropathic pain syndromes
• Centrally sensitised pain conditions

All patients had previously failed or experienced inadequate response to standard medical management and complementary pain therapies.

### 2.5. Exclusion Criteria

Patients were excluded from IVL infusion if any of the following were present:

• Patient refusal or inability to provide informed consent
• Chronic pain duration of less than 6 months
• Known cardiac arrhythmia, including atrial fibrillation or other clinically significant dysrhythmias
• Significant structural heart disease
• Epilepsy or uncontrolled neurological disorders
• Known hypersensitivity to amide‐type local anaesthetics
• Previous documented lack of response to IVL infusion

Baseline safety assessment included review of medical history, medication profile and preinfusion electrocardiogram (ECG) to exclude arrhythmia or conduction abnormalities.

### 2.6. Procedure and Infusion Protocol

All patients undergoing IVL infusion completed the WHO Surgical Safety Checklist prior to treatment. The risks, benefits and potential adverse effects of IVL were explained in detail, and written informed consent was obtained from all participants.

### 2.7. Preprocedure Assessment

All patients underwent a structured preprocedure assessment in the chronic pain clinic prior to scheduling IVL infusion, to confirm clinical indication and ensure eligibility according to predefined inclusion and exclusion criteria. For patients who had previously undergone IVL infusion, a detailed assessment of prior response was conducted.

On the day of treatment, patients were assessed according to a standardised protocol. This included confirmation of informed consent, comprehensive review of medical history and current medications and documentation of baseline physiological parameters, including heart rate, blood pressure and oxygen saturation. A baseline ECG was performed in all cases to exclude arrhythmias or conduction abnormalities.

Fasting status was verified in accordance with institutional anaesthetic guidelines at University Hospitals of Leicester, requiring a minimum fasting period of 6 h for solid food. For clear fluids, there was no strict restriction, and patients were permitted to take small sips of water up until transfer to the procedure area. This was undertaken to minimise the risk of nausea and vomiting associated with intravenous infusion therapy.

All infusions were administered in a monitored day‐case theatre environment in accordance with local Trust protocols, ensuring continuous physiological monitoring and immediate access to resuscitation facilities.

### 2.8. Infusion Administration

Intravenous access was established, and lidocaine was administered at a standard dose of 3 mg/kg (ideal body weight) over 1 h using a syringe pump. Continuous monitoring was performed throughout the infusion in accordance with Association of Anaesthetists guidance, including

• Continuous electrocardiography
• Noninvasive blood pressure monitoring
• Pulse oximetry

Patients remained under observation during the infusion and for a monitored recovery period afterwards. Discharge occurred only after patients met standard clinical safety and discharge criteria, including haemodynamic stability, absence of significant adverse effects and return to baseline neurological status.

### 2.9. Number and Frequency of Infusions

The number of IVL infusions offered was individualised based on clinical response and patient‐reported benefit. Approximately 25 patients per month were treated within the service. Over the study period of approximately six months, this corresponded to 136 patients, taking into account service variability, including weekends, public holidays and reduced activity during holiday periods.

Patients who demonstrated meaningful clinical improvement were considered for repeat infusion. Clinical decisions regarding repeat treatment were based on

• Duration of analgesic benefit
• Functional improvement
• Reduction in analgesic medication use
• Overall patient satisfaction

There was no predefined maximum number of infusions, provided that patients continued to derive clinical benefit and met safety criteria.

In routine practice, repeat infusions were planned at approximately 6‐month intervals where clinically indicated. However, due to significant service demand and prolonged waiting times, actual intervals between treatments were frequently extended beyond 9 months.

### 2.10. Data Collection

Patient data were retrospectively obtained from electronic hospital systems, including ORMIS, Nervecentre, ICE and Dictate 3.

Collected variables included

• Demographic information
• Pain diagnosis
• Duration of symptoms
• Previous treatments
• Infusion details
• Documented adverse events
• Repeat infusion history

### 2.11. Follow‐Up Arrangements

Patients were routinely contacted by telephone at approximately 3 months post‐infusion by an Advanced Nurse Practitioner in Pain Medicine and invited to complete a structured assessment. During this follow‐up, patients were asked to retrospectively recall their symptom response, duration of benefit, functional improvement and overall experience during the preceding post‐infusion period. A further clinical review was typically undertaken at 6 months by a pain physician.

The follow‐up assessment evaluated

• Numerical pain score
• Subjective degree of pain improvement
• Duration of analgesic effect
• Functional improvement
• Quality of life
• Patient satisfaction
• Presence and type of adverse effects
• Reduction in analgesic medication use

Quality of life and functional outcomes were assessed using standardised clinical parameters, including return to work or usual activities, ability to perform activities of daily living and overall well‐being and mood. All responses were documented in the electronic medical record. For patients undergoing repeat infusions, prior response, duration of benefit, functional improvement and safety profile were reviewed to inform subsequent treatment decisions.

### 2.12. Governance and Safety

The IVL service operates within a consultant‐led tertiary pain pathway under University Hospitals of Leicester NHS Trust protocol. Administration occurs in a monitored clinical environment with established safety procedures and predefined exclusion criteria to minimise risk of cardiac or neurological complications. Patients receiving IVL infusion at Leicester General Hospital between October 2024 and March 2025 were identified (*n* = 136). All patients were eligible for follow‐up survey.

**FIGURE 1:**
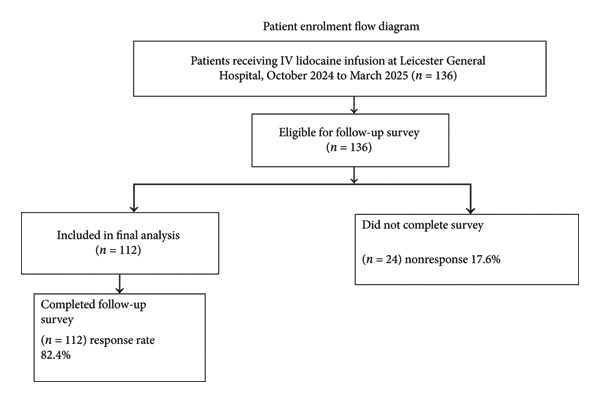
Patient enrolment flowchart.

Of these, 112 patients (82.4%) completed the follow‐up survey and were included in the final analysis, while 24 patients (17.6%) did not complete the survey and were classified as nonresponders. The response rate of 82.4% is acceptable; however, the possibility of response bias remains, as patients who derived greater benefit may have been more likely to complete follow‐up, potentially leading to overestimation of treatment effect (see Figure 1).

## 3. Results

### 3.1. Study Population

A total of 136 patients received IVL infusion during the study period. Of these, 112 patients completed follow‐up assessment, corresponding to a response rate of 82.4%. Only respondents were included in outcome analysis.

### 3.2. Demographic and Clinical Characteristics

There was a marked female predominance, with 93 patients (83%) being women and 19 (17%) men. The majority of patients were aged between 40 and 60 years (61 patients, 54.5%), while 51 patients (45.5%) were either younger than 40 years or older than 60 years (Table 1).

**TABLE 1:** Baseline characteristics of respondents (*n* = 112).

Variable	Category	*n* (%)
Sex	Female	93 (83)
	Male	19 (17)

Age group	40–60 years	61 (54.5)
	< 40 or > 60 years	51 (45.5)

Previous IVL infusion	Yes	58 (52)
	No	54 (48)

The most common indications for IVL infusion were fibromyalgia, chronic neuropathic pain, chronic myofascial pain and trigeminal neuralgia.

A total of 58 patients (52%) had previously received IVL infusion. Within this subgroup, the majority had undergone more than three prior infusions, suggesting perceived clinical benefit and acceptability of the treatment. However, as prior lack of response was an exclusion criterion, this represents a selection bias towards patients who had previously derived benefit, potentially influencing overall outcome estimates.

Baseline pain characteristics were documented at initial assessment. Patients typically reported moderate‐to‐severe chronic pain, with high baseline pain scores and significant impact on function, daily activities and overall quality of life. A comparison between patients receiving their first infusion and those with prior exposure was considered; however, this was limited by sample size and retrospective data completeness.

### 3.3. Treatment Outcomes

#### 3.3.1. Pain Intensity Reduction

Baseline pain intensity, measured using the Numerical Rating Scale (NRS, 0–10), was high across the cohort, with a mean baseline score of 8.0 (range: 10–6). At 2 months post‐infusion, the mean NRS decreased to 4.5 (range: 6–3), representing an absolute reduction of 3.5 points. This exceeded the widely accepted threshold for clinically meaningful improvement, defined as either a ≥ 2‐point absolute reduction or ≥ 30% reduction in NRS score.

A pain score threshold of ≤ 4.5 was used pragmatically to define sustained or prolonged response, reflecting transition from severe to moderate pain severity and aligning with clinically meaningful functional improvement in chronic pain populations.

**FIGURE 2:**
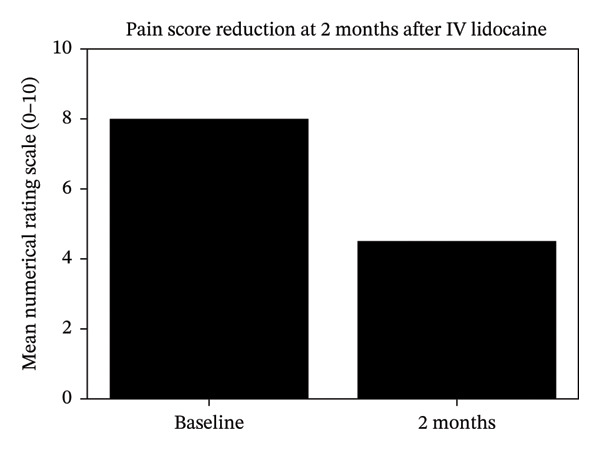
Pain score after 2 months of IV lidocaine infusion (*n* = 112).

**FIGURE 3:**
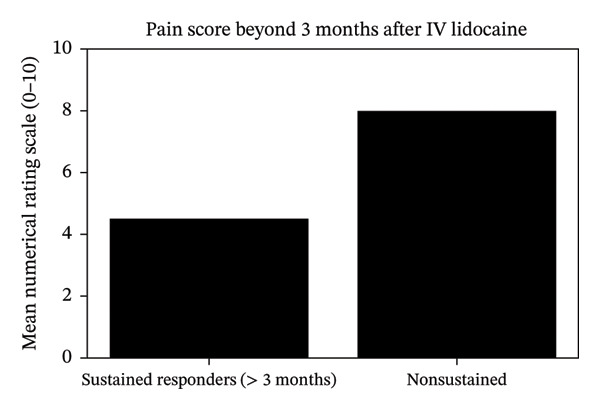
Average pain score (NRS) after 3 months (*n* = 112).

At 2 months, 96 patients (86%) reported sustained improvement compared with baseline. Long‐term benefit, defined as maintenance of pain scores ≤ 4.5 beyond 3 months, was observed in 32 patients (29%) within the cohort (Figures 2 and 3).

#### 3.3.2. Duration of Analgesic Benefit

**FIGURE 4:**
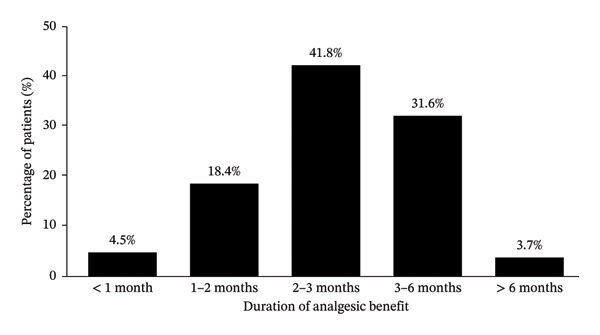
Duration of analgesic benefit following IV lidocaine infusion (*n* = 112).

The distribution of duration of pain relief is shown in Figure 4.

• Less than 1 month: 4.5%
• 1‐2 months: 18.4%
• 2‐3 months: 41.8%
• 3–6 months: 31.6%
• Greater than 6 months: 3.7%

Approximately 60% of patients reported benefit lasting between 2 and 3 months (Figure 4).

#### 3.3.3. Adverse Events

Adverse effects were reported by 19 patients (17%). All reported side effects were mild and self‐limiting. Among patients reporting side effects, the most frequently reported symptoms were

• Tiredness (45%)
• Dizziness (25%)
• Lethargy (20%)
• Pain flare‐up (20%)
• Transient hypotension (5%)
• Palpitations (5%)
• Tingling sensations (5%)

**FIGURE 5:**
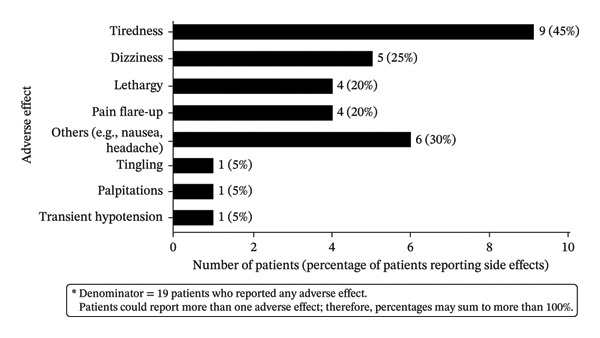
Distribution of adverse effects among patients reporting side effects following IV lidocaine infusion (*n* = 19)^∗^.

No serious adverse events were observed. In particular, no arrhythmias or seizures were reported (Figure 5).

#### 3.3.4. Patient Satisfaction

**FIGURE 6:**
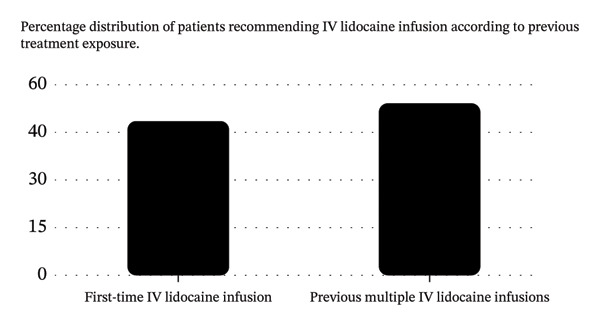
Recommendation of IV lidocaine infusion according to previous exposure.

Overall satisfaction with treatment was high. Ninety‐five patients (85%) indicated that they would recommend IVL infusion for chronic pain management. Of these, 45 patients (approximately 47%) were receiving IVL infusion for the first time, while 50 patients (approximately 53%) had previously undergone more than one IVL infusion (Figure 6).

## 4. Discussion

This retrospective service evaluation examined patient‐reported outcomes following IVL infusion within a monitored tertiary chronic pain service. In this cohort, the majority of patients reported improvement in pain intensity following IVL infusion, with mean NRS decreasing from 8.0 to 4.5 during the early posttreatment period, representing a clinically meaningful reduction in pain severity. Sustained analgesic benefit beyond 3 months was observed in 29% of patients, although overall durability of effect was variable.

While the magnitude of short‐term pain reduction was clinically significant, the duration of benefit varied considerably. The majority of patients reported analgesia lasting between two and three months, with fewer patients experiencing prolonged benefit beyond this period. It is important to note that duration of analgesic effect was derived from patient recall during a single follow‐up assessment, typically conducted at approximately 3 months. As such, long‐term estimates are subject to recall bias, particularly for durations extending beyond several months, and should be interpreted as approximate rather than precise time‐to‐event outcomes.

These findings are consistent with published literature suggesting that IVL provides short‐term neuromodulatory analgesia rather than sustained long‐term disease modification [1, 2, 16, 17]. The biological rationale for IVL use in chronic pain is well established. Lidocaine inhibits voltage‐gated sodium channels, suppresses ectopic discharges from injured peripheral nerves and may modulate central sensitisation processes [3, 13]. Early experimental work demonstrated abnormal spontaneous activity in injured afferent neurons following peripheral nerve injury, supporting the mechanistic basis for sodium channel blockade in neuropathic pain [13]. Clinical investigations have shown variable analgesic responses across chronic pain conditions [4–7, 17].

Systematic reviews and meta‐analyses report modest but heterogeneous short‐term benefit, particularly in neuropathic pain syndromes [1, 2, 16, 17]. Randomised trials have demonstrated short‐term efficacy but inconsistent long‐term benefit [18]. Evidence synthesis highlights substantial variability in dosing, outcome measures and follow‐up duration, limiting comparability across studies [1, 2].

Emerging studies have explored repeated or serial infusions, suggesting potential for extended benefit in selected contexts [8, 12, 19]. In our cohort, repeat infusions were planned at approximately 6‐month intervals; however, service demand and prolonged waiting times frequently extended intervals beyond 9–12 months, limiting the number of repeat treatments during the study period. Consequently, only a small proportion of patients underwent repeat infusions, restricting the ability to assess cumulative or sustained effects. Furthermore, treatment decisions for repeat infusion were informed by prior response, duration of benefit, functional improvement and safety profile, introducing selection bias towards patients who had previously derived benefit.

The inclusion of heterogeneous pain conditions reflects real‐world tertiary practice but limits condition‐specific inference. While previous studies have reported IVL use across a range of pain syndromes [7–11, 14, 15], our study did not include stratified or phenotype‐based analysis and therefore cannot support conclusions regarding phenotype‐dependent efficacy. IVL should be considered an adjunctive therapy with variable response across chronic pain conditions, rather than a universally effective intervention.

Safety outcomes in this study were favourable and consistent with existing literature [4, 6, 18, 20]. Adverse effects were reported in 17% of patients, were mild and self‐limiting and occurred during or shortly after infusion. Importantly, the reported distribution of adverse effects reflects the frequency within the subgroup of patients experiencing side effects (*n* = 19) rather than overall population prevalence. No serious complications, including arrhythmias or seizures, were observed. The structured protocol, including preinfusion ECG screening and continuous monitoring, likely contributed to this favourable safety profile.

Patient satisfaction was high, with 85% of patients indicating willingness to recommend treatment. While this aligns with the proportion of patients receiving repeat infusions, it should be interpreted with caution. The inclusion of patients with prior positive response may have contributed to overestimation of satisfaction, and the voluntary nature of survey participation introduces potential response bias, whereby patients experiencing greater benefit may have been more likely to respond.

This study has several strengths. It represents a relatively large, real‐world cohort within a structured NHS tertiary pain service, with a high follow‐up response rate. The monitored day‐case delivery model enhances safety and reproducibility. However, several important limitations must be acknowledged. The retrospective design and reliance on a single time‐point follow‐up introduce recall and reporting bias. Outcomes were patient‐reported and not prospectively standardised using validated multidimensional instruments. Functional outcomes and quality of life were assessed using pragmatic clinical parameters (e.g., return to activity and general well‐being), rather than validated scales. Additionally, the use of a pragmatic NRS threshold (≤ 4.5) to define sustained response was not derived from validated outcome instruments and should be interpreted cautiously. The absence of a control group precludes causal inference and limits differentiation between treatment effect, placebo response and regression to the mean. Finally, the single‐centre design may limit generalisability.

Future research should prioritise prospective, controlled study designs with predefined longitudinal follow‐up at multiple time points, incorporation of validated outcome measures and standardisation of treatment protocols. Such studies would reduce bias, improve accuracy in estimating duration of effect and better define the role of IVL within chronic pain pathways.

## 5. Conclusion

IVL infusion appears to be a safe and generally well tolerated option for selected patients with refractory chronic pain, particularly neuropathic pain, fibromyalgia and myofascial pain syndromes. In this study, the majority of patients reported meaningful pain improvement, with a subset experiencing benefit lasting longer than 3 months. Reported adverse effects were mild and self‐limiting, and overall patient satisfaction was high.

The high rate of repeat infusions within the service suggests perceived benefit and acceptability among patients. However, given the retrospective design and reliance on patient reported outcomes, these findings should be interpreted with caution. While IVL may provide short‐ to medium‐term analgesic benefit, definitive conclusions regarding sustained long‐term efficacy cannot be drawn from this dataset alone.

## Funding

The authors declare that this research received no external funding.

## Ethics Statement

All procedures performed in studies involving human participants were conducted in accordance with the ethical standards of the institutional and/or national research committee and with the 1964 Helsinki Declaration and its later amendments or comparable ethical standards.

## Conflicts of Interest

The authors declare no conflicts of interest.

## Data Availability

The data that support the findings of this study are available from the corresponding author upon reasonable request.
